# Novel Copper-Zinc-Manganese Ternary Metal Oxide Nanocomposite as Heterogeneous Catalyst for Glucose Sensor and Antibacterial Activity

**DOI:** 10.3390/antiox11061064

**Published:** 2022-05-27

**Authors:** Mir Waqas Alam, Hassan S. Al Qahtani, Basma Souayeh, Waqar Ahmed, Hind Albalawi, Mohd Farhan, Alaaedeen Abuzir, Sumaira Naeem

**Affiliations:** 1 Al Bilad Bank Scholarly Chair for Food Security in Saudi Arabia, The Deanship of Scientific Research, The Vice Presidency for Graduate Studies and Scientific Research, King Faisal University, Al-Ahsa 31982, Saudi Arabia; bsouayeh@kfu.edu.sa (B.S.); mfarhan@kfu.edu.sa (M.F.); aabuzir@kfu.edu.sa (A.A.); 2 Department of Physics, College of Science, King Faisal University, Al-Ahsa 31982, Saudi Arabia; 3 EXPEC Advanced Research Centre, Saudi Aramco, Dhahran 31311, Saudi Arabia; hassan.alqahtani.2@aramco.com; 4 Takasago i-Kohza, Malaysia-Japan International Institute of Technology, Universiti Teknologi Malaysia, Kuala Lumpur 54100, Malaysia; waqarum.ah@gmail.com; 5 Department of Physics, College of Sciences, Princess Nourah bint Abdulrahman University (PNU), P.O. Box 84428, Riyadh 11671, Saudi Arabia; 6 Department of Basic Sciences, Preparatory Year Deanship, King Faisal University, Al-Ahsa 31982, Saudi Arabia; 7 Department of Chemistry, University of Gujrat, Gujrat 50700, Pakistan; sumaira.usman@uog.edu.pk

**Keywords:** absorption, glucose, methylene blue, sensor, trimetal oxide

## Abstract

A novel copper-zinc-manganese trimetal oxide nanocomposite was synthesized by the simple co-precipitation method for sensing glucose and methylene blue degradation. The absorption maximum was found by ultraviolet–visible spectroscopy (UV-Vis) analysis, and the bandgap was 4.32 eV. The formation of a bond between metal and oxygen was confirmed by Fourier Transform Infrared Spectroscopy (FT-IR) analysis. The average crystallite size was calculated as 17.31 nm by X-ray powder diffraction (XRD) analysis. The morphology was observed as spherical by scanning electron microscope (SEM) and high-resolution transmission electron microscopy (HR-TEM) analysis. The elemental composition was determined by Energy Dispersive X-ray Analysis (EDAX) analysis. The oxidation state of the metals present in the nanocomposites was confirmed by the X-ray photoelectron spectroscopy (XPS) analysis. The hydrodynamic diameter and zeta potential of the nanocomposite were 218 nm and −46.8 eV, respectively. The thermal stability of the nanocomposite was analyzed by thermogravimetry-differential scanning calorimetry (TG-DSC) analysis. The synthesized nanocomposite was evaluated for the electrochemical glucose sensor. The nanocomposite shows 87.47% of degradation ability against methylene blue dye at a 50 µM concentration. The trimetal oxide nanocomposite shows potent activity against *Escherichia coli*. In addition to that, the prepared nanocomposite shows strong antioxidant application where scavenging activity was observed to be 76.58 ± 0.30, 76.89 ± 0.44, 81.41 ± 30, 82.58 ± 0.32, and 84.36 ± 0.09 % at 31, 62, 125, 250, and 500 µg/mL, respectively. The results confirm the antioxidant potency of nanoparticles (NPs) was concentration dependent.

## 1. Introduction

Presently, diabetes is considered one of the most common chronic diseases and is already declared a serious threat to a healthy lifestyle [1]. Several medical conditions are associated with diabetes, including heart diseases, tuberculosis, coeliac disease, and cystic fibrosis. These diseases can result in blindness and nephropathy that can even cause renal failure, nerve damage, foot ulcers, and cancer [2,3]. An abnormal level of glucose in the human body is a major source of diabetes, which comes from daily food products such as potatoes, fruit, and preservative food. Consuming food rich in glucose levels can increase blood sugar and, in turn, cause diabetes [4,5]. Therefore, due to daily increasing risks to health, it is important to develop a highly sensitive and simple technique to detect and monitor glucose levels for clinical diagnosis, medicine, and the food industry. In addition to that, the detection of glucose levels is also specifically important in several food products because glucose can cause browning due to dehydration and longer storage time. Moreover, monitoring glucose is also highly important in pharmaceutical products where several food products are being used [6]. Until now, metal oxides (MOs) and metal sulfides (MSs) have been used as advanced electrocatalysts for developing highly sensitive electrochemical sensors [7,8,9]. However, these sensors have drawbacks such as low detection limits [10,11,12]. Due to excellent electrical and chemical properties, several nanomaterials including metal oxides, nanoparticles, graphene, and carbon nanotubes are frequently used as electrochemical biosensors [13]. It has also been reported that a combination of two or several nanomaterials as nanocomposites further enhance these properties and are frequently used in biosensing applications [14]. High-performance ternary metal oxide nanocomposites such as ZnO/MnO_2_/Cu_2_O [15], ZnO/Fe_2_O_3_/MnO_2_ [16], and ZnO/MnO_2_/Gd_2_O_3_ [17] have been reported for enhanced photocatalytic application. For the effective removal of organic contaminants, heterogenous photocatalysis is considered an important class of advanced oxidation processes [18]. To enhance the photocatalytic properties and glucose sensing, the ternary nano heterojunction combination with a compatible valence band can highly useful. Similarly, a Fe/La/Zn nanocomposite with graphene oxides was prepared by another group of researchers to analyze the photodegradation of phenylhydrazine, environmental remediation, and antimicrobial studies [19]. The undiscriminating usage of antimicrobial/antibacterial drugs for treating various infectious diseases has also attracted the attention of researchers to develop organic and inorganic nanomaterial agents. Extreme oxidative sensitivity and instability issues related to organic antibacterial nanoparticles pose a limitation to their usage and simultaneously suggest crossing this barrier by using inorganic materials for this purpose based on their capability to tolerate severe procedural conditions. Metal oxides are considered among the best choices to prepare antimicrobial medicines owing to their stability as well as their safety for humans, animals, and plants [20]. For example, the synthesis of one-dimensional (1D) MgO nanowires via the microwave hydrothermal method has gained remarkable attention in preparing antimicrobial medicines due to their enriched surface chemistry with special emphasis on the high surface area [21]. Silicon nanowires decorated with silver nanoparticles are also reported as synthetic one-dimensional semiconducting long-term antibacterial nanomaterials, which have been gaining interest in recent years [22]. Similarly, a green approach to biosynthesizing silver nanowires from commercial *Camellia sinesis* has been considered for their antibacterial properties against *Escherichia coli* and *Staphylococcus aureus*, which proved fatal for infectious microorganisms [23]. Nanocomposites, especially heterocyclic ones, are considered advantageous over metallic nanoparticles as they play the role of a junction between two or more nanoparticles due to their high versatility. They have a promising position in medicinal chemistry owing to their wide spectrum of activities [24], including photosensitizer (e.g., Ag/Ti/Zn Trimetallic Nanoparticles and Carbon Quantum Dots Nanocomposites) [25], antimicrobial [26], and antioxidant activity (e.g., bio-Ag/CdO/ZnO@MWCNTs nanocomposites) [27], by using several different methods. For antibacterial activities, zinc- and copper-doped TiO_2_ nanoparticles have been reported. The results show that the doping of both the materials is considered an essential antibacterial agent, and strongly influenced photocatalytic degradation and antibacterial properties [28,29]. In addition, Polyvinylchloride coated with a combination of silver nanoparticles and zinc oxide nanowires also showed potential for antimicrobial applications [30].

Because of their high surface area, biocompatibility, and excellent electrical and catalytic properties, Cu-Zn-Mn nanocomposites are proven to be highly efficient sensors [31,32,33]. Due to the compatible band structure and narrow bandgap of Cu, Zn, and Mn, they are highly useful for photocatalytic applications [34]. Several methods, such as biosynthesis, the hydrothermal route, and screen-printing, have been used to prepare the ternary nanocomposite [35]. However, the co-precipitation method is a frequently used technique for developing a nanocomposite of mixed oxides. The application of the co-precipitation method in the current research area has shown a significant difference in the phase of calcined oxides with mixed nanocomposites [36].

Therefore, in the current study, the author aims to produce a copper-zinc-manganese ternary metal oxide nanocomposite as a heterogeneous catalyst via the co-precipitation method. The synthesized nanocomposite was evaluated for an electrochemical glucose sensor, photocatalytic degradation of methylene blue, and antioxidant and antibacterial applications.

## 2. Experimentation

### 2.1. Materials

The precursors, such as copper sulphate pentahydrate—CuSO_4_·5H_2_O (99%), zinc nitrate hexahydrate—Zn(NO_3_)_2_·6H_2_O (99%), and manganese acetate tetrahydrate—Mn(CH_3_COO)_2_·4H_2_O (99%) used for trimetal oxide nanomaterial synthesis, were purchased from Sigma Aldrich (St. Louis, MO, USA). An aqueous ammonia solution was used as the precipitating agent. D-glucose was used for glucose-sensing applications. Methylene blue was chosen as a pollutant for the degradation process. Double-distilled water was used as a solvent throughout the experiments.

### 2.2. Methods

#### Synthesis of Cu-Zn-Mn Mixed Trimetal Oxide

**Figure 1:**
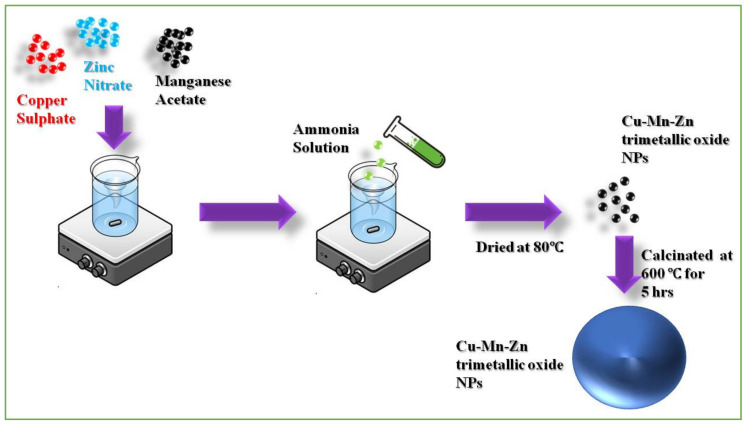
Synthesis of Cu-Zn-Mn mixed trimetal oxide nanoparticles (NPs).

In a typical reaction process, 1 mM copper sulphate pentahydrate was dissolved in 30 mL of double-distilled water and stirred for 15 min. To that, 1 mM manganese acetate tetrahydrate in 30 mL of double-distilled water was added, and stirring was continued for 45 min. To the above solution, 1 mM zinc nitrate hexahydrate in 30 mL of double-distilled water was added and stirring was continued for one hour. To the above precursor solutions, 15 mL of an aqueous ammonia solution was added, and the pH was maintained at 12. The black-colored precipitates were obtained and washed via the centrifugation process several times with a 3:1 ratio of water and the ethanol mixture. Then the precipitates were dried at 80 °C and calcinated at 600 °C for 5 h. The black-colored precipitates were obtained as a final product and were stored for further characterization and application processes. Figure 1 depicts the synthesis of Cu-Zn-Mn mixed trimetal oxide nanoparticles.

### 2.3. Characterization

The crystalline phase, structure, and size of the synthesized trimetal oxide were found by X-ray powder diffraction (XRD) analysis performed with an Ultima IV Rigaku diffractometer (Rigaku, Tokyo, Japan) with Cu Kα as a radiation source (λ = 1.540 Å, 40 kV, 15 mA) and it was recorded in the range of 10–90°. The morphology and average particle size of the material were analyzed by SEM with EDX (Bruker, Billerica, MA, USA) at an operating voltage of 15 kV. The atomic and weight percentages were found using EDX analysis. XPS was performed using the PHI Versaprobe III (ULVAC-PHI, Inc., Chanhassen, MN, USA), which was recorded in the range of 0 to 1100 eV. HR-TEM analysis was performed using JEOL-2100 (JEOL Ltd., Tokyo, Japan). The FT-IR spectrometer (Jasco Inc., Tokyo, Japan) was used to determine the functional groups present in the nanocomposite and confirm the formation of a bond between metal and oxygen. The FT-IR analysis was recorded in the wavenumber range of 4000–400 cm^−1^ with KBr as a standard and a scanning speed of 2 cm^−1^/s. The particle size and zeta potential were found using Dynamic Light Scattering (DLS) analysis. The synthesized nanomaterials were dispersed by the sonication process for 20 min in an ultrasonic bath and analyzed directly using a DLS instrument (Horiba, Tokyo, Japan) and the temperature was set to 25 °C during the analysis. The graphite electrode was used to perform and measure the zeta potential of the synthesized nanoparticles. The optical property was investigated by UV-Vis analysis using a JASCO spectrophotometer (Jasco Inc., Tokyo, Japan) and recorded in the range of 200–800 nm with a scanning speed of 1000 nm/min, and the bandgap was found using Tauc’s plot. The thermal stability of the synthesized trimetal oxide nanocomposite was confirmed using the TG-6300 analyzer (Seiko Instruments Inc., Tokyo, Japan) under a nitrogen atmosphere.

### 2.4. Electrochemical Measurement

Electrochemical measurement was carried out for the glucose sensor application at room temperature. The standard three-electrode system (working electrode-prepared carbon paste electrode, reference electrode-Ag/AgCl electrode, and indicator electrode-platinum wire) was used for the electrochemical tests. 1 M KOH solution was used as an electrolyte and D-glucose was used as an analyte for glucose sensor measurement.

### 2.5. Photocatalytic Degradation of Dye Solution

The photocatalytic degradation of methylene blue (MB) dye as a pollutant was carried out under sunlight as an irradiation source in the presence of trimetal oxide as a photocatalyst. The degradation study was carried out in a batch reactor system. Briefly, 250 mL of the dye solution was taken in a borosilicate rectangular tray. The tray contained a known concentration (50 µM) of MB solution and 100 mg of the photocatalyst. After the addition of the photocatalyst, the pH of the solution was maintained at 7 ± 0.1 by adding the NaOH solution. Later, the solution was allowed to stir for 30 min using a magnetic stirrer in a dark room to attain the adsorption–desorption equilibrium of MB on the catalyst surface. The degradation of MB was carried out under direct sunlight and the intensity of the light was frequently measured. At particular time intervals, the degraded solution was continuously collected. The residual concentration of MB dye solution was measured at 662 nm using a UV-Vis spectrophotometer (Jasco Inc., Tokyo, Japan). The intensity of the sunlight was measured as 840–860 lux using a digital lux meter. The same protocol was followed for different concentrations, such as 75 and 100 µM dye solution.

### 2.6. Antioxidant Activity

#### DPPH Scavenging Activity

The antioxidant potency of the Cu-Zn-Mn mixed trimetal oxide nanocomposite was determined using a 1,1-diphenyl-2-picrylhydrazyl radical (DPPH) assay by following the protocols of [37]. To carry out the measurement, 100 µg of the Cu-Zn-Mn mixed trimetal oxide nanocomposite was mixed with 100 µL of ethanol and 50 µL of the DPPH solution and kept in a dark room for 30 min. The absorption value was observed at 518 nm for the prepared solution. Ascorbic acid was used as a standard antioxidant agent. Antioxidant activity was determined by calculating the percent (%) of inhibition by the following formula (Equation (1)):

Scavenging effect (%) = [(A_c_ − A_s_)/A_c_] × 100 (1)

where A_c_ is the absorbance of the control and A_s_ is the absorbance of the sample.

### 2.7. Antibacterial Activity

The antibacterial efficacy of synthesized NPs was investigated against the pathogen *Escherichia coli*. The assessment of antibacterial activity was confirmed using the agar well diffusion method by adopting the protocols of (Burygin et al., 2009) with slight modifications [38].

## 3. Results and Discussion

### 3.1. XRD Analysis

**Figure 2:**
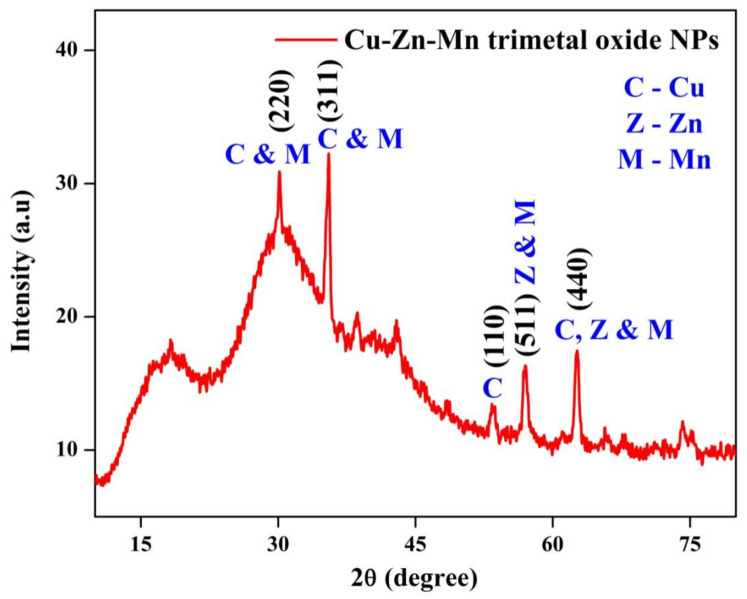
XRD pattern of Cu-Zn-Mn mixed trimetal oxide nanoparticles.

The XRD pattern for the Cu-Zn-Mn mixed trimetal oxide nanocomposite is given in Figure 2. From the analysis, the 2θ value was observed at 30.19, 35.48, 53.42, 56.95, and 62.51°. Compared with JCPDS card No. (83-1665), the peak observed at 30.19, 35.48, 53.42, and 62.51° corresponded to the miller indices (200), (202), (314), and (020) for CuO and had a tetragonal phase. The peaks observed at 56.95 and 62.51° correspond to the miller indices (110) and (103), which confirmed the formation of ZnO, and it had a hexagonal phase. The formation of ZnO was confirmed by the JCPDS card No. 89-1397. According to JCPDS card No. 86-2337, the formation of MnO_2_ was confirmed by the peaks at 30.19, 35.48, 56.95, and 62.51° with respect to the miller indices (011), (201), (341), and (112), and it had an orthorhombic phase. The average crystalline size of synthesized nanomaterial was found to be 17.31 nm. Juma et al. synthesized the CuO-NiO-ZnO mixed metal oxide, and XRD analysis was carried out. At a calcined temperature of 200 °C, the CuO and ZnO could be identified and NiO was not identified, which may be due to the Ni being in its hydroxide form. At a calcined temperature of 200 °C, CuO, ZnO, and NiO were well resolved, which confirmed that all the hydroxides were decomposed and converted to oxides [39].

### 3.2. SEM-EDAX Analysis

**Figure 3:**
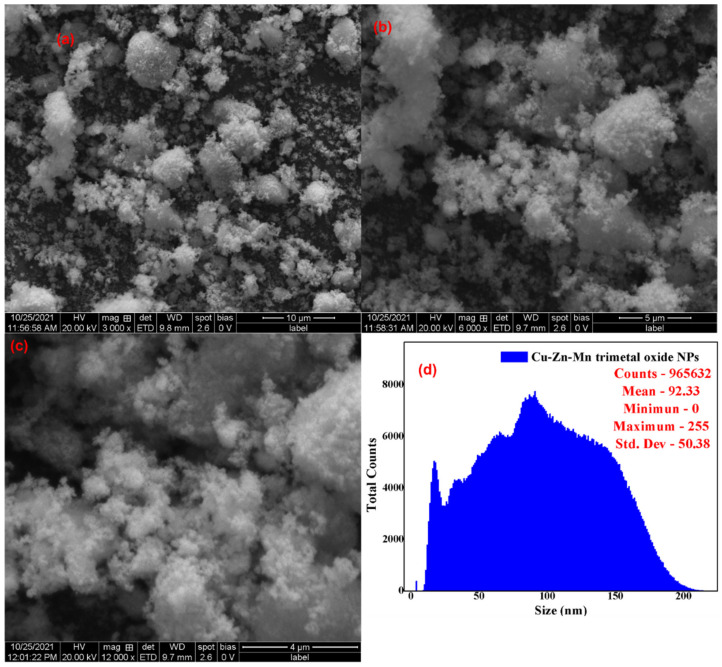
SEM of Cu-Zn-Mn mixed trimetal oxide nanoparticles. (**a**) 10 µm; (**b**) 5 µm; (**c**) 4 µm; (**d**) size distribution.

Figure 3 shows the SEM analysis of the Cu-Zn-Mn mixed trimetal oxide nanocomposite (a) with 10 µm, (b) with 5 µm, (c) with 4 µm, and (d) the size distribution. The SEM analysis indicates the synthesized nanoparticle was non-uniformly spherical in morphology with some aggregation, which leads to enhancement of the particle size. The average particle size was found to be 92.33 nm with a total count of 965,632 particles with the aid of ImageJ software. The trimetal oxide has shown the agglomeration of particles, which is usual for all types of nanomaterials due to the involvement of strong interparticle contact-induced utilizing high surface energy. Enhessari et al. synthesized aggregated quasi-spherical CuMn_2_O_4_ nanoparticles with an average particle size of 39 nm [40]. According to Sanchez et al., the aggregation of the nanoparticles was reduced by increasing the reaction time [41].

**Figure 4:**
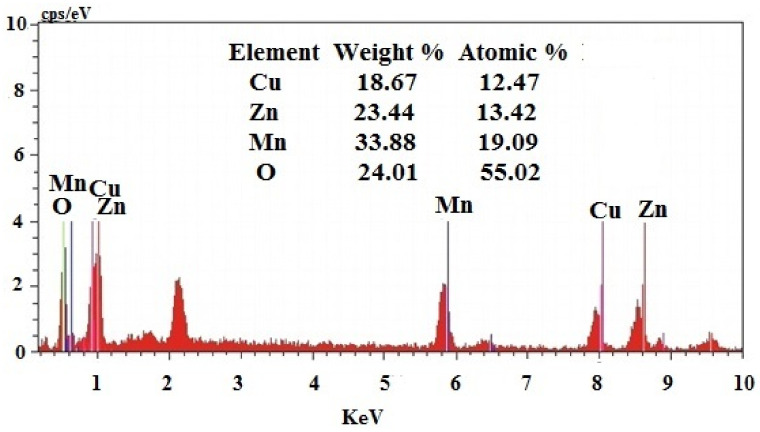
EDAX of Cu-Zn-Mn mixed trimetal oxide nanoparticles.

The atomic and weight percentage of the synthesized nanoparticle was found by using EDAX analysis. Figure 4 shows the EDAX of the Cu-Zn-Mn mixed trimetal oxide nanocomposite. The weight % age of Cu, Zn, Mn, and O was found to be 18.67, 23.44, 33.88, and 24.01, respectively. The atomic % age of Cu, Zn, Mn, and O was found to be 12.47, 13.42, 19.09, and 55.02, respectively. The error % of Cu, Zn, Mn, and O was found to be 0.43, 0.64, 0.87, and 2.96, respectively.

### 3.3. HR-TEM—SAED Analysis

**Figure 5:**
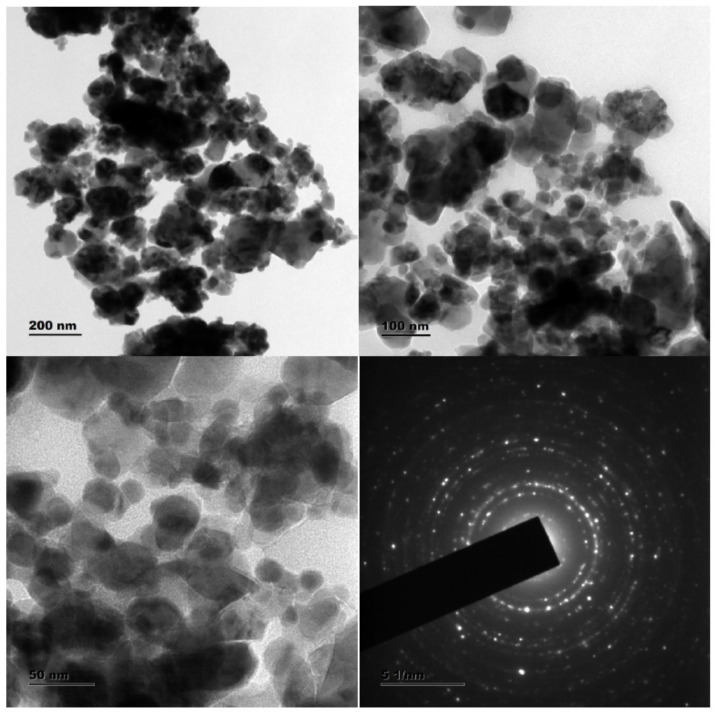
HR-TEM—SAED analysis of Cu-Zn-Mn mixed trimetal oxide nanoparticles.

The surface morphology of the synthesized Cu-Zn-Mn mixed trimetal oxide was analyzed by HR-TEM analysis (Figure 5). HR-TEM revealed that synthesized nanoparticles are spherical in morphology with agglomeration. Compared with XRD, the particle size was higher in HR-TEM (90 ± 3 nm), which may be due to the merging of particles to form larger particles. This shows that the formed particles are agglomerated, which is elucidated by the HR-TEM images of lattice fringes. The Selected area diffraction (SAED) model illustrates bright rings that prove the superior orientation of nanocrystals. The SAED pattern contains many rings with different radii, which shows that the Cu-Zn-Mn mixed trimetal oxide nanoparticles belong to a nanocrystalline structure. The diffraction rings matched with XRD results and the rings from inside to outside can be indexed to (220), (311), (110), (511), and (440) crystal planes.

### 3.4. XPS Analysis

**Figure 6:**
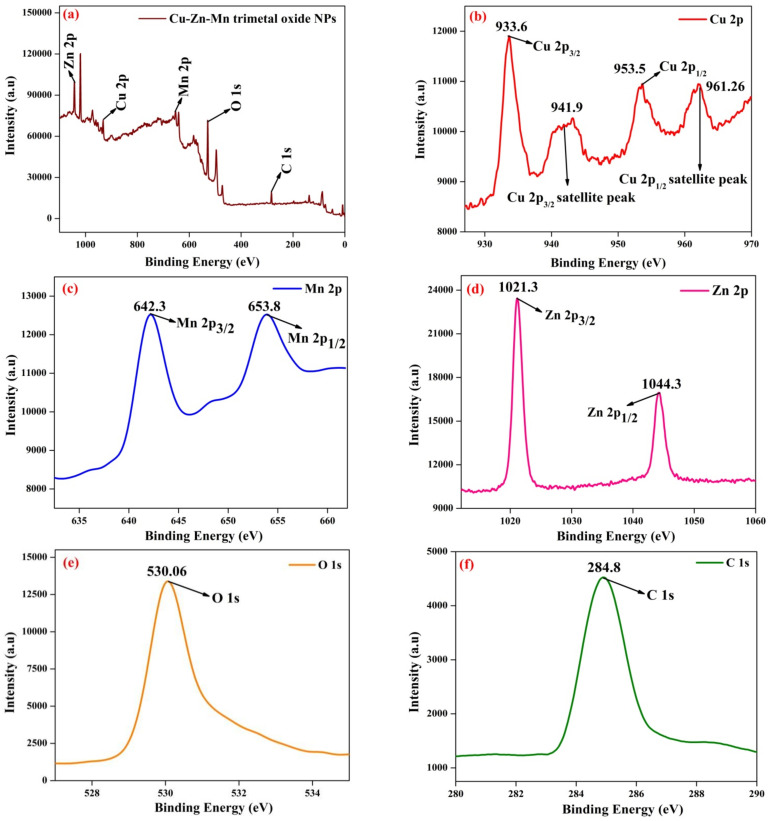
XPS of Cu-Zn-Mn mixed trimetal oxide nanoparticles. (**a**) survey scan of trimetal oxide; (**b**) Cu, (**c**) Mn, (**d**) Zn, (**e**) O, (**f**) C elements.

The elemental composition and oxidation state of Cu-Zn-Mn mixed trimetal oxide nanocomposites were determined using XPS analysis in accordance with the binding energies of the various elements present on the material’s surface, and the XPS spectrum was recorded between 0 and 1100 eV. Figure 6a shows the XPS survey scan of the Cu-Zn-Mn mixed trimetal oxide revealed the existence of Cu, Zn, Mn, O, and C. Figure 6b shows the Cu 2p core level consists of Cu 2p_3/2_ and Cu 2p_1/2_ at 933.6 eV and 953.5 eV, respectively, and it can be readily notified in the high-resolution spectrum of Cu 2p. At the same time, two characteristic satellite peaks at 941.90 eV and 961.26 eV can be clearly noticed, indicating the presence of Cu 3p [42]. Figure 6c shows the Mn 2p core level consists of Mn 2p_3/2_ and Mn 2p_1/2_ at 642.3 eV and 653.7 eV, respectively [43]. The characteristic peak appeared at 1021.3 eV and 1044.3 eV with respect to Zn 2p_3/2_ and Zn 2p_1/2_, respectively, for the Zn 2p core level region (Figure 6d) [44]. In the case of the O 1s core level (Figure 6e), the signal can be attributed to oxygen molecules attached to the different atoms. The peak at approximately 530.6 eV is ascribed to metal-oxygen bonds [45]. In the core level of C 1s (Figure 6f), the peak at approximately 284.8 eV is ascribed to the C-C bond based on the literature value [46].

### 3.5. DLS Analysis

**Figure 7:**
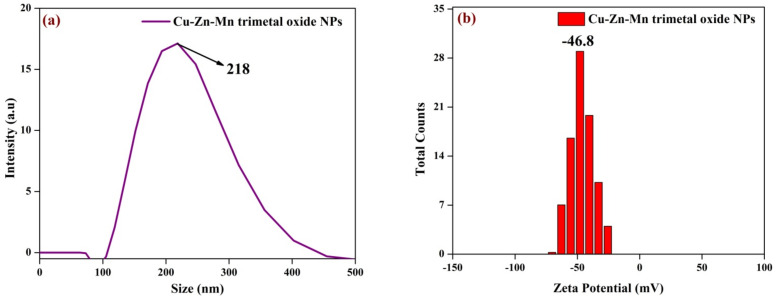
(**a**) Particle size and (**b**) Zeta potential of Cu-Zn-Mn mixed trimetal oxide nanoparticles.

The hydrodynamic diameter of the Cu-Zn-Mn mixed trimetal oxide nanocomposite was found to be 218 nm. Figure 7a,b shows the DLS analysis and zeta potential of Cu-Zn-Mn mixed trimetal oxide nanoparticles, respectively. The surface charge of the synthesized trimetal oxide was measured by zeta potential. The surface charge of the particle indicates the stability of trimetal oxide nanocomposite, which was found to be −46.8 mV. The zeta potential value in the range of ˃±30 mV shows the nanomaterial has sufficient repulsive force to attain better physical colloidal stability, and the zeta potential range of ±40 to 50 mV shows the nanomaterial has good stability [47].

### 3.6. UV-Vis Analysis

**Figure 8:**
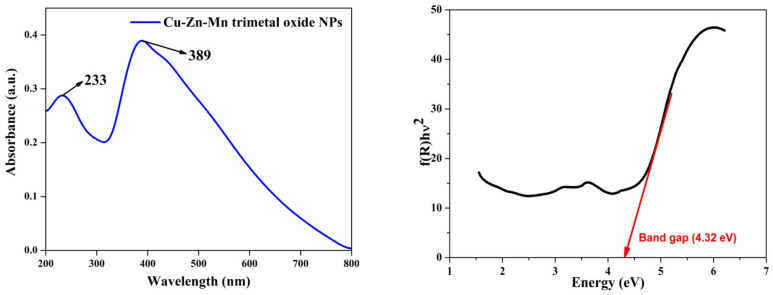
UV-Vis and Tauc plot of Cu-Zn-Mn mixed trimetal oxide nanoparticles.

Figure 8 shows the UV-Vis spectrum of the Cu-Zn-Mn mixed trimetal oxide nanocomposite. The two peaks were observed in the UV region at wavelengths of 233 nm and 389 nm and these peaks correspond to the metal oxide. The two peaks observed in the spectrophotometer were owing to the interband fundamental edge transitions. The absorbance value moved towards the visible region, which was due to the presence of ZnO in the synthesized trimetal oxide nanocomposite. Similarly, copper-doped ZnO nanoparticles have an absorbance peak at 230 and 270 nm, which confirms the formation of a metal oxide. After doping the ZnO with Cu, the intensity of the absorbance peak increased, which was due to the zinc ions present in the crystal lattices leading to the alteration, especially in the extension of lattices [48]. Khalid et al. synthesized zinc-doped copper oxide nanoparticles, and the absorbance peak was observed at 390 nm with zinc doping, which was due to the d–d transition [49]. John Abel synthesized CuMn_2_O_4_ nanoparticles and the absorbance value was observed at 278 nm [50]. The optical bandgap of the Cu-Zn-Mn mixed oxide nanocomposite was found by Tauc’s plot. From the Tauc relation, the bandgap of the synthesized nanocomposite was found to be 4.32 eV, which confirmed that the synthesized nanoparticle was a semiconductor in nature. The Tauc relation is shown in Equation (2):

(αhυ) α (hυ − E_g_)^n^ (2)

### 3.7. FT-IR Analysis

**Figure 9:**
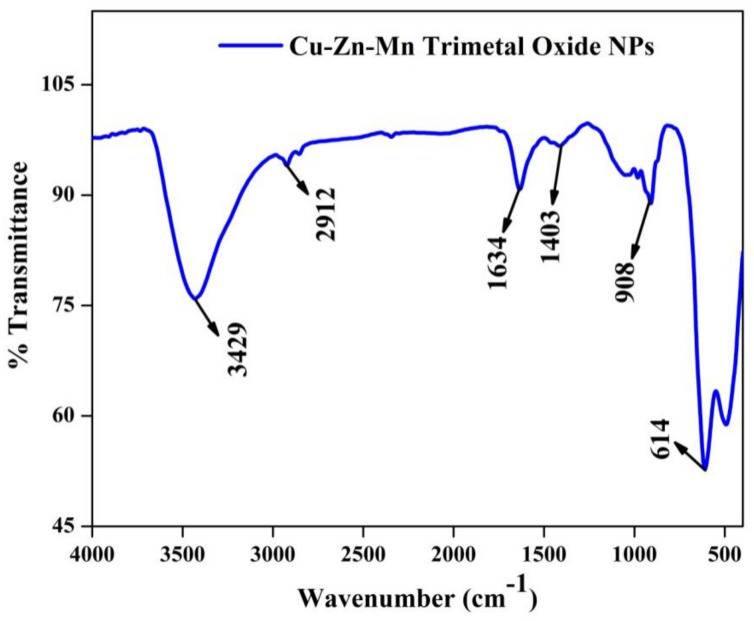
FT-IR of Cu-Zn-Mn mixed trimetal oxide nanoparticles.

The chemical bonding and functional group present in the synthesized Cu-Zn-Mn mixed-metal oxide nanoparticle was found using FT-IR analysis. Figure 9 shows the FT-IR analysis of the Cu-Zn-Mn mixed oxide nanocomposite. The band at 3429 cm^−1^ was assigned to the O-H stretching vibration, which was due to the moisture content present on the surface of the nanocomposite [51]. The C-H stretching vibration was confirmed by the peak at 2912 cm^−1^. The peak appeared at 1634 cm^−1^ accredited to O-H bending vibration in the nanocomposite. The peak that appeared at 1403 cm^−1^ was attributed to the carboxy C-O stretching vibration [52]. The presence of C=C bending vibration present in the nanocomposite was confirmed by the peak that appeared at 908 cm^−1^. The formation of the bond between metal and oxygen during nanoparticle formation was found by the peak appearing at 614 cm^−1^ [53].

### 3.8. TG-DSC Analysis

**Figure 10:**
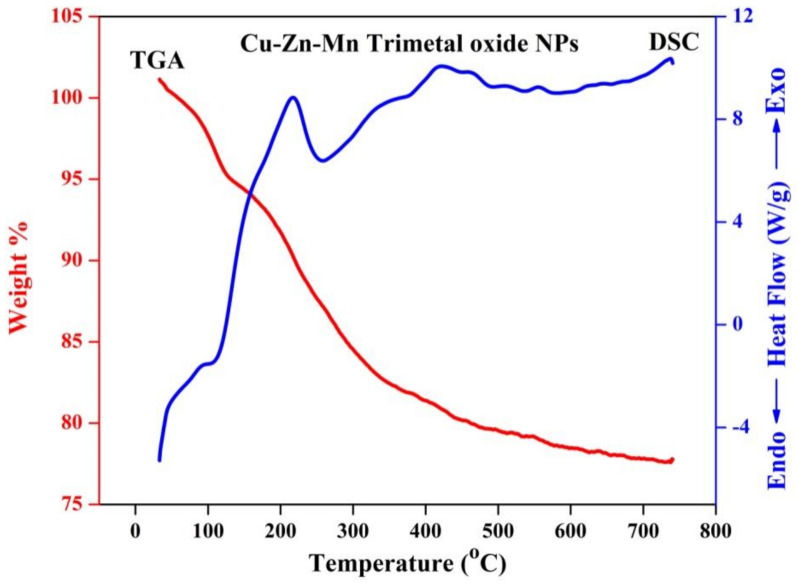
TG-DSC of Cu-Zn-Mn mixed trimetal oxide nanoparticles.

Figure 10 depicts the thermogravimetric analysis (TGA) and Differential scanning calorimetry (DSC) analysis of Cu-Zn-Mn trimetal oxide nanocomposite. The decomposition temperature of the synthesized material was found to be 67–148 and 436 °C, corresponding to a weight loss of 5.87 and 19.52%. The DSC curve illustrates the overlap of exothermic and endothermic peaks, approximately in the range of 200–270 °C. An endothermic peak was observed at 216 °C, and this might be due to the surface reaction initiated among metal nanoparticles. An exothermic peak was found at 258 °C, which may be due to the latent heat of crystallization, stabilization of crystal structure, and diffusion rearrangement of unstable atoms [54]. The removal of water molecules from the synthesized material was confirmed by the decomposition temperature in the range of 67–148 °C, which was observed from TGA analysis. Before 200 °C, the weight of synthesized nanoparticles steadily decreased while increasing the temperature without noticeable exothermic and endothermic peaks, which shows that there was no chemical reaction taking place. The peak at 436 °C was due to the decomposition of anions, which shows the conversion of hydroxide to the corresponding metal oxide nanoparticles, which was reliable considering the result of XRD. At the continuous temperature rise, there was no noticeable weight loss observed, which indicates that the nanoparticle was thermally stable after 450 °C, and 80% of the sample remained.

### 3.9. Glucose Sensor

**Figure 11:**
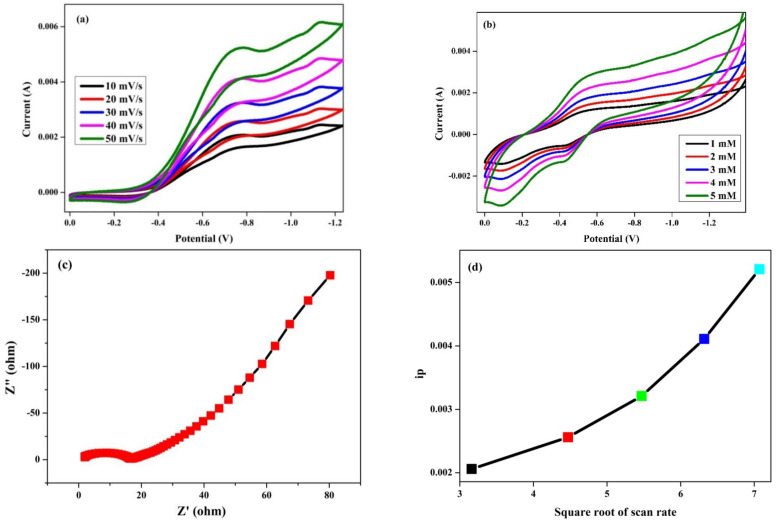
(**a**) CV graph of Cu-Zn-Mn mixed trimetal oxide nanoparticles with different scan rates, (**b**) CV graph with glucose at different concentrations, (**c**) Nyquist plots of the Cu-Zn-Mn mixed trimetal oxide, (**d**) plot of peak current vs. square root of scan rate.

Figure 11a shows the cyclic voltammetry (CV) of mixed trimetal oxide nanoparticles. The CV graph without glucose shows there is no proper appearance of peaks in oxidation, but during the reduction, the appearance of a small peak was observed at −0.7 V. During the sensing of glucose, oxidation and reduction peaks appeared. From Figure 11a, it is seen that, with an increased scan rate, the corresponding current peaks are increased; meanwhile, the oxidation peaks observed shifted slightly toward the positive side, and the reduction peaks shifted slightly towards the negative side of the potential. Figure 11b shows the CV results at different concentrations (1–5 mM) of the glucose analyte. A significant difference was observed between the cathodic and anodic peak intensities, which suggests the prepared materials have strong sensing applications [55]. By increasing the concentration of the analyte, a significant increase in the peak intensity was observed. As per these results, it is clearly seen that the prepared trimetal oxide nanoparticles are highly suitable for glucose sensing. As shown in Figure 11c, electrochemical impedance spectroscopy (EIS) studies were also investigated for the synthesized trimetal oxide nanoparticles to clearly recognize the resistance mechanism. The presence of a smaller area of the semi-circle for the electrode reveals that the ohmic resistance offered by the electrode in the basic medium is low due to its higher capacitance. For confirmation of the electrode process, Figure 11d shows the peak current vs. the square root of the scan rate where we can clearly observe a linear line, which indicates a diffusion-controlled reaction.

### 3.10. Photocatalytic Degradation of MB Dye

**Figure 12:**
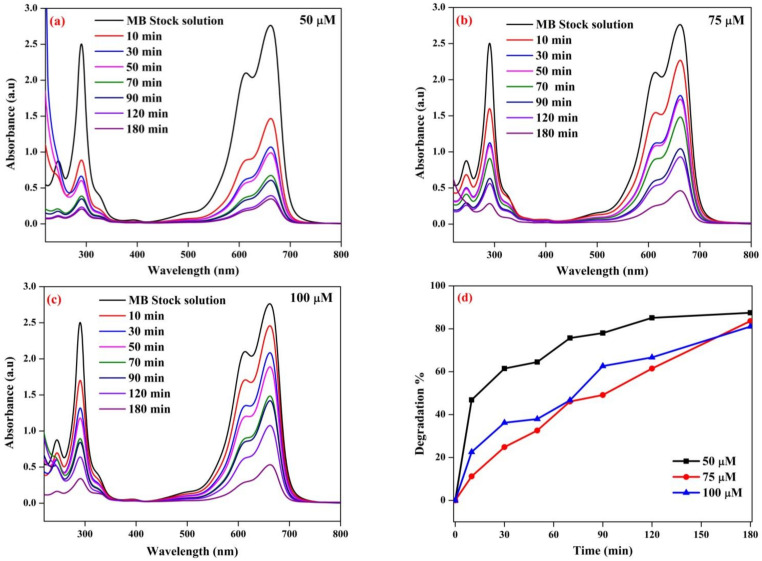
Photocatalytic degradation of methylene blue at (**a**) 50 µM, (**b**) 75 µM, and (**c**) 100 µM. (**d**) Degradation efficiency of Cu-Zn-Mn trimetal oxide NPs.

The photocatalytic degradation efficiency of the synthesized Cu-Zn-Mn trimetal oxide nanocomposite was investigated against methylene blue dye as a pollutant and the reaction was carried out at neutral pH. The degraded solution was collected at different time intervals and tested using a UV-Vis spectrophotometer. Upon the degradation of the dye, three bands were observed at 661, 288, and 244 nm. Figure 12a–c shows the degradation of MB corresponding to concentrations of 50, 75, and 100 µM, respectively. The band observed at 661 nm in the visible region corresponds to a sulfur-nitrogen conjugated system. The intensity of the band gradually diminished as the degradation progressed, and this confirmed that the sulfur-nitrogen conjugated system was damaged during the degradation process. The color of the MB solution was slowly lightened during the constant irradiation of sunlight. The bands at 244 and 288 nm in the UV region indicate the phenothizine group present in the MB. This is owing to the oxidative decomposition and ring-opening reaction in phenothizine species. Photocatalysis generates a strong oxidation agent to break organic matter into CO_2_ and H_2_O in the presence of light and photocatalysts [56]. The degradation efficiency of nanoparticles was found to be 87.47, 83.6, and 81.02% with respect to 50, 75, and 100 µM. Figure 12d illustrates the degradation efficiency of synthesized nanoparticles. The % degradation was calculated using the following formula (Equation (3)).

Degradation% = [(A0 − At)/A0] × 100(3)

**Figure 13:**
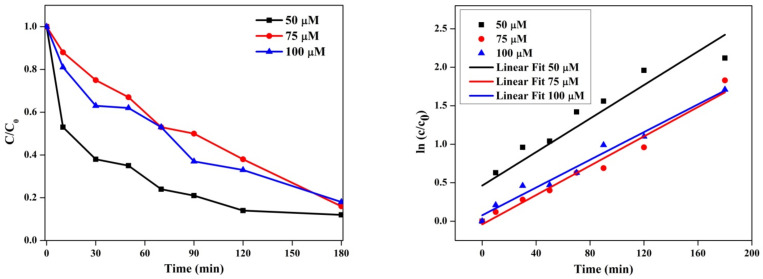
Plot of time vs. c/c_0_ and time vs. ln c/c_0_ for photocatalytic degradation of methylene blue dye.

The absorbance value was decreased with the increased time of solar irradiation with the photocatalyst. The intensity of the blue color faded after the degradation of the dye solution. The degradation of dye was possible due to the generation of hole (h^+^) and electron (e^−^) pairs on the surface of the catalyst during the irradiation of sunlight. The e^−^ and h^+^ pair interacts with a water molecule to produce a hydroxyl radical, which is responsible for breaking the hazardous dye constituents. Electrons present in the conduction band on the catalyst’s surface convert molecular oxygen to superoxide ions and, finally, forms hydrogen peroxide. The hole produces a hydroxyl radical, which plays a key role in the breakage of dye molecules. The efficiency of the photocatalyst decreased with the increasing concentration of the dye. The efficiency was indirectly proportional to the concentration of the dye [57]. The rate constant (k) corresponding to 50, 75, and 100 µM was found to be 0.01089, 0.00953, and 0.009 and the linear regression coefficient (R^2^) corresponding to 50, 75, and 100 µM was found to be 0.8554, 0.9661, and 0.9758 (Figure 13).

#### Regeneration and Reusability

**Figure 14:**
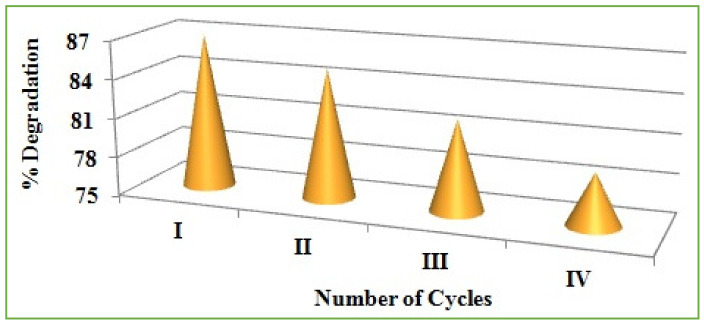
Reusability of catalyst for methylene blue degradation.

The regeneration and reusability of the photocatalyst are very important to evaluate the efficiency of the catalyst. After completion of the degradation process, the catalyst was collected from the reaction medium and further used for reusability study. To collect the catalyst, the degraded solution was centrifuged at 3000 rpm and the supernatant solution was discarded. Then the precipitate was thoroughly washed, thrice, with a 3:1 water:ethanol mixture to remove the pollutant. The obtained precipitate was washed and dried at 80 °C to remove the moisture content and impurities present in the catalyst. The collected catalyst underwent three reusability studies, and the efficiency of the catalyst was diminished during the studies. The efficiency % was reduced from 87 to 79 % after four cycles. This is due to the leaching of the catalyst during washing and drying. The weight of the catalyst was decreased, which leads to a decrease in the efficiency of the catalyst. Figure 14 illustrates the reusability of the catalyst for MB dye degradation.

### 3.11. Antioxidant Activity

**Figure 15:**
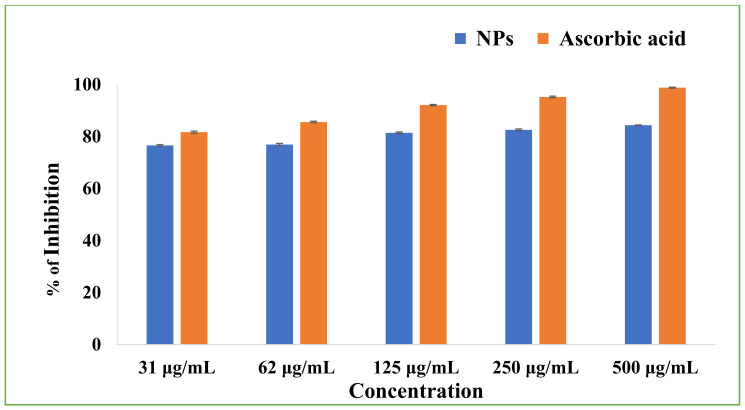
DPPH scavenging activity of Cu-Zn-Mn mixed trimetal oxide nanoparticles.

To determine the antioxidant potency of the Cu-Zn-Mn mixed trimetal oxide nanocomposite, ascorbic acid was used as a standard for DPPH assay. The scavenging activity of the NPs was increased with increasing concentrations of NPs. The scavenging activity was observed to be 76.58 ± 0.30, 76.89 ± 0.44, 81.41 ± 30, 82.58 ± 0.32, and 84.36 ± 0.09 % at 31, 62, 125, 250, and 500 µg/mL, respectively (Figure 15). The study confirmed that the antioxidant potency of NPs was concentration dependent.

### 3.12. Antibacterial Activity

**Figure 16:**
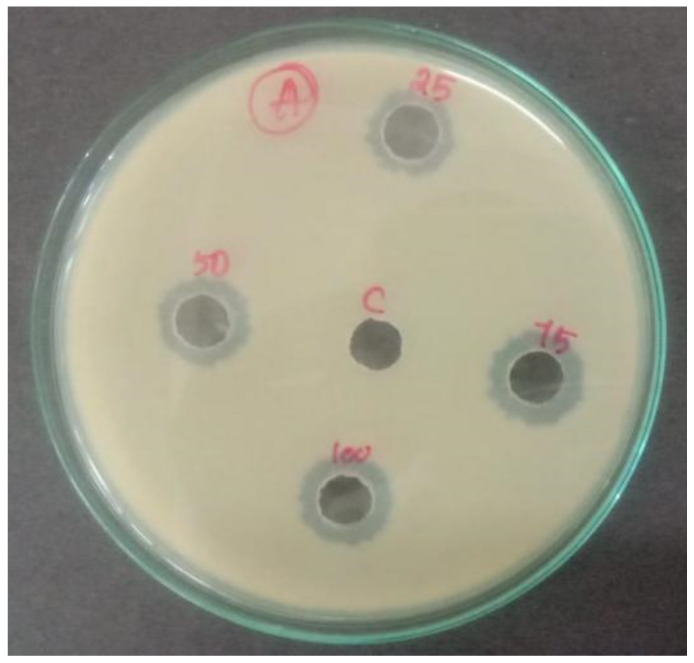
Antibacterial activity of Cu-Zn-Mn mixed trimetal oxide nanoparticles.

The results of the study suggested that the synthesized NPs showed potential antibacterial effects against *E. coli*. Figure 16 depicts the antibacterial activity of Cu-Zn-Mn mixed trimetal oxide nanoparticles against *E. coli.* The minimum and maximum zones of inhibition were noted as 12 mm and 16 mm at 25 and 100 µg/mL concentrations, respectively. At 50 and 75 µg/mL concentrations, the zone of inhibition was noted as 13.5 and 14.6 mm, respectively. Hence, it is clearly evidenced that the synthesized NPs for antibacterial activity were concentration-dependent and have the potency to inhibit the growth of pathogenic bacterial strains. The Gram-negative bacteria have an outer membrane made up of negatively charged lipopolysaccharide molecules. It seems that the synthesized nanomaterial passes through the cell wall of the bacteria, and it is damaged from the interior. The physical interaction took place between the bacterial cells and synthesized NPs, which creates the cell wall structure disruption, leading to the breakdown and, finally, bacterial death [58].

## 4. Conclusions

In summary, the Cu-Zn-Mn mixed trimetal oxide NPs were successfully synthesized, characterized, and investigated for a glucose sensor, photocatalytic degradation of methylene blue, and antioxidant and antibacterial activity. The synthesized nanoparticles were spherical with an average particle size of 92 nm. The semiconductor nature was confirmed by the bandgap value of 4.32 eV. The nanoparticles were thermally stable, confirmed by TG-DSC analysis. The hydrodynamic diameter and zeta potentials were 218 nm and −46.8 mV. The binding energy of the elements was found by XPS analysis. The degradation efficiency of the photocatalyst was found to be 87% against methylene blue. The antibacterial and antioxidant activity of the nanoparticles were concentration-dependent. The nanoparticles were active for glucose sensor application.

## Data Availability

Data is contained within this article.
